# Revisitando uma Competência Intemporal: O Exame Físico na Era da Medicina Moldada Pela Tecnologia

**DOI:** 10.36660/abc.20250124

**Published:** 2025-04-15

**Authors:** Sofia Cabral

**Affiliations:** 1 Centro Hospitalar Universitário do Porto Serviço de Cardiologia Porto Portugal Serviço de Cardiologia, Centro Hospitalar Universitário do Porto, Porto - Portugal; 2 Universidade do Porto Instituto de Ciências Biomédicas Abel Salazar Porto Portugal Instituto de Ciências Biomédicas Abel Salazar, Universidade do Porto, Porto - Portugal

**Keywords:** Exame Físico, Tomada de Decisão Clínica, Educação Médica

Nas últimas décadas, a prática clínica testemunhou uma transformação profunda, impulsionada pela crescente utilização de imagens avançadas, diagnósticos laboratoriais e, mais recentemente, ferramentas assistidas por inteligência artificial (IA). Antes a pedra angular da prática médica, o exame físico (EF) agora está sendo ofuscado por uma crescente dependência da tecnologia. Embora essas inovações tenham, sem dúvida, melhorado a precisão do diagnóstico, elas também levaram a uma consequência não intencional — o declínio gradual das competências do exame clínico à cabeceira do doente, à medida que os médicos confiam cada vez mais nelas em vez do EF direto. Embora esses recursos forneçam *insights* diagnósticos inestimáveis, eles também carregam riscos, incluindo falsos positivos, achados incidentais e testes excessivos. Esses fatores não apenas contribuem para intervenções desnecessárias e aumento dos custos de saúde, mas também podem amplificar as preocupações e ansiedade do doente. Essa mudança da avaliação prática também é parcialmente impulsionada pelas restrições de tempo na abordagem clínica contemporânea, enfraquecendo ainda mais as competências no EF.^1^ Este declínio do EF tem alimentado um debate contínuo — e até mesmo polarização — entre os médicos sobre sua relevância na prestação atual de cuidados médicos.^2 , 3^ Essa questão permeia todos os campos da medicina, e a cardiologia não é exceção aos seus desafios.^4^

Em seu estudo, Teixeira et al.^5^ lançam luz sobre esta questão premente na medicina moderna: a atenuação da importância do EF na prática clínica. Este estudo intervencionista avaliou o impacto do EF cardiovascular na tomada de decisão clínica (TDC) em cenários de doença valvar. Sessenta estudantes de graduação foram expostos a quatro cenários típicos de doença valvar grave (estenose mitral [EM], regurgitação mitral [RM], estenose aórtica [ES], e regurgitação aórtica [RA]), usando um simulador cardiopulmonar de alta fidelidade em um formato de exame clínico estruturado objetivo. As perguntas relacionadas à TDC foram respondidas após a leitura da sinopse clínica e novamente após a revisão do relatório do ecocardiograma (ECO). Os voluntários foram aleatoriamente designados para cenários com ou sem EF antes de receber um relatório do ECO, que era consistente com os sintomas e achados do EF (Eco-concordante) ou continha informações incompletas ou enganosas (Eco-discordante). A TDC foi avaliada com base nas respostas sobre o tipo de disfunção valvar, sua etiologia, nível de confiança no diagnóstico, solicitações de testes diagnósticos adicionais e decisões de tratamento. A qualidade do EF variou entre as condições valvares, com sopros sistólicos (ES, RM) reconhecidos com mais precisão (> 76%) do que sopros diastólicos (EM, RA) (< 60%), sons cardíacos adicionais (S3, S4) identificados em 44% dos casos e EM mostrando a menor precisão de detecção. A precisão diagnóstica geral para identificar disfunção valvar foi alta (kappa = 0,935, p < 0,001), mesmo quando os participantes receberam relatórios discordantes do ECO. Os voluntários que não realizaram EF foram menos precisos na avaliação da gravidade da disfunção valvar após a revisão dos achados do ECO (p = 0,0047, kappa = 0,2887) e estavam menos confiantes em seu diagnóstico. O nível de confiança no diagnóstico aumentou apenas ligeiramente (4%, p = 0,03) após receber os relatórios do ECO naqueles que tiveram a oportunidade de conduzir um EF. Surpreendentemente, um alto número de exames complementares foi solicitado independentemente da realização de EF, que influenciou apenas a decisão de realizar o cateterismo cardíaco. As decisões terapêuticas finais, no entanto, foram guiadas principalmente pelos achados do ECO em vez do EF (p = 0,0607). Este último resultado era um tanto esperado e provavelmente ocorreria também em ambientes de prática médica efetiva. De fato, ao avaliar a necessidade de intervenção valvar, uma consideração importante — além do estado sintomático — é determinar a gravidade da doença, que depende principalmente de parâmetros de imagem ou hemodinâmicos.^6 - 8^

A cardiologia moderna evoluiu para uma disciplina em que a TDC requer a integração da avaliação junto ao doente com tecnologias de diagnóstico sofisticadas, dentro de uma estrutura clínica abrangente. O valor clínico do EF na cardiologia carece de evidência consistente, e este estudo fornece *insights* valiosos.^5 , 9^ Os achados de Teixeira et al.^5^ levam-nos a reconsiderar o papel do EF na educação médica pré-graduada, com implicações que se estendem ao treino pós-graduado.

Por outro lado, os rápidos avanços em modalidades de imagem, como ecocardiografia, ressonância magnética cardíaca e tomografia computadorizada cardíaca, juntamente com diagnósticos baseados em biomarcadores e algoritmos aprimorados por IA, melhoraram significativamente a precisão diagnóstica, a estratificação de risco e o desenvolvimento de estratégias terapêuticas personalizadas. Essas inovações não apenas melhoram a sobrevivência do doente, mas também impulsionam o progresso na medicina cardiovascular contemporânea. Sua capacidade de detectar doenças em estágios iniciais, quantificar a gravidade com precisão sem precedentes e orientar intervenções oportunas remodelou fundamentalmente a TDC no campo das doenças cardiovasculares.^10^

No entanto, a dependência excessiva dessas tecnologias corre o risco de enfraquecer o raciocínio clínico e as competências junto do doente, ao mesmo tempo em que contribui para a desumanização da prática e amplia as disparidades no acesso a diagnósticos avançados. Além disso, além do seu valor diagnóstico, essas competências promovem confiança, comunicação e sintonia entre doentes e clínicos, elementos essenciais da tomada de decisão compartilhada e do cuidado centrado no doente.

Para abordar isso, o treino médico deve enfatizar uma abordagem equilibrada, garantindo que os clínicos integrem *insights* tecnológicos com um julgamento clínico sólido para uma melhor gestão clínica do doente. Para conseguir isso, o treinamento de EF —independentemente do formato de ensino—e de outras competências junto do doente devem ser reavaliadas e receber maior importância.^11^ O EF continua sendo uma ferramenta clínica fundamental que, além de sua contribuição inestimável para a compreensão da fisiopatologia, pode auxiliar na tomada de decisões, aumentar a eficiência e fortalecer o relacionamento médico-doente.

Em última análise, em vez de ver os avanços tecnológicos e as competências clínicas junto do doente como forças opostas, a comunidade médica deve se esforçar para ter uma perspectiva integrada, livre de concepções dogmáticas sobre os méritos e as deficiências de cada um. Reinventar o EF na educação médica e na prática diária garantirá que os clínicos aproveitem o melhor dos dois mundos — alavancando a tecnologia moderna enquanto preservam o valor insubstituível da avaliação prática.
