# Supplementary material for: Revisitando uma Competência Intemporal: O Exame Físico na Era da Medicina Moldada Pela Tecnologia
Source: Arq Bras Cardiol. 2025 Apr 15;122(2):e20250124. [Article in Portuguese] doi: 10.36660/abc.20250124 (PMC12094291; doi:10.36660/abc.20250124)
Supplement: Translation

# Translation: Revisiting a Timeless Skill: The Physical Examination in the Age of Technology-Driven Medicine

**DOI:** 10.36660/abc.20250124i

**Authors:** Sofia Cabral

**Affiliations:** 1 Porto Portugal Serviço de Cardiologia, Centro Hospitalar Universitário do Porto, Porto - Portugal; 2 Porto Portugal Instituto de Ciências Biomédicas Abel Salazar, Universidade do Porto, Porto - Portugal

**Keywords:** Physical Examination, Clinical Decision-Making, Medical Education

Over the past few decades, clinical practice has witnessed a profound transformation, driven by increasing reliance on advanced imaging, laboratory diagnostics, and, more recently, artificial intelligence (AI)-assisted tools. Once the cornerstone of medical practice, the physical examination (PE) is now being overshadowed by a growing dependence on technology. Although these innovations have undoubtedly enhanced diagnostic accuracy, they have also led to an unintended consequence—the gradual decline of bedside examination skills, as clinicians increasingly rely on them instead of direct PE. While these resources provide invaluable diagnostic insights, they also carry risks, including false positives, incidental findings, and overtesting. These factors not only contribute to unnecessary interventions and rising healthcare costs but may also amplify patient concerns. This shift away from hands-on assessment is also partly driven by the time constraints of modern clinical practice, further weakening PE skills.^1^This decline of PE has fuelled ongoing debate—and even polarization—among clinicians over its relevance in contemporary healthcare.^2 , 3^This issue permeates all fields of medicine, and cardiology is no exception to its challenges.^4^

In their study, Teixeira et al.^5^shed light on this pressing issue in modern medicine: the diminished emphasis on PE in clinical practice. This interventional study evaluated the impact of cardiovascular PE on clinical decision-making (CDM) in valvular disease scenarios. Sixty undergraduate students were exposed to four typical severe valvular disease scenarios (mitral stenosis [MS], mitral regurgitation [MR], aortic stenosis [AS], and aortic regurgitation [AR]), using a high-fidelity cardiopulmonary simulator in an objective structured clinical exam format. CDM-related questions were answered after reading the clinical synopsis and again after reviewing the echocardiogram (ECHO) report. Volunteers were randomly assigned to scenarios with or without PE before receiving an ECHO report, which was either consistent with symptoms and PE findings (Echo-concordant) or contained incomplete or misleading information (Echo-discordant). CDM was assessed based on responses regarding the type of valvular dysfunction, its etiology, confidence level in the diagnosis, requests for additional diagnostic tests, and treatment decisions. PE quality varied across valvular conditions, with systolic murmurs (AS, MR) recognized more accurately (>76%) than diastolic murmurs (MS, AR) (<60%), additional heart sounds (S3, S4) identified in 44% of cases, and MS showing the lowest detection accuracy. Overall diagnostic accuracy for identifying valvular dysfunction was high (kappa = 0.935, p<0.001), even when participants received ECHO-discordant reports. Volunteers who did not perform PE were less accurate in assessing the severity of valvular dysfunction after reviewing ECHO findings (p = 0.0047, kappa = 0.2887) and were less confident in their diagnosis. The confidence level in diagnosis increased only slightly (4%, p=0.03) after receiving ECHO reports in those who had the opportunity to conduct a PE. Surprisingly, a high number of complementary exams were requested regardless of PE performance, which influenced only the decision to perform cardiac catheterization. Final therapeutic decisions, however, were primarily guided by ECHO findings rather than PE (p=0.0607). This last result was somewhat expected and would likely occur in a postgraduate setting as well. Indeed, when evaluating the need for valvular intervention, a key consideration—besides symptomatic status—is assessing disease severity, which primarily depends on imaging or hemodynamic parameters.^6 - 8^

Modern cardiology has evolved into a discipline where CDM requires the integration of both bedside assessment and sophisticated diagnostic technologies within a comprehensive clinical framework. The clinical value of PE in cardiology lacks consistent evidence, and this study provides valuable insight.^5 , 9^Teixeira et al.^5^findings compel us to reassess the role of PE in undergraduate medical education, with implications extending to postgraduate training.

Conversely, the rapid advancements in imaging modalities such as echocardiography, cardiac magnetic resonance imaging, and cardiac computed tomography, along with biomarker-based diagnostics and AI-enhanced algorithms, have significantly improved diagnostic precision, risk stratification and the development of tailored therapeutic strategies. These innovations not only improve patient survival but also drive progress in contemporary cardiovascular medicine. Their ability to detect disease at earlier stages, quantify severity with unprecedented accuracy, and guide timely interventions has fundamentally reshaped CDM in the field of cardiovascular disease.^10^

However, over-reliance on these technologies risks weakening clinical reasoning and bedside skills while also contributing to the dehumanization of practice and widening disparities in access to advanced diagnostics. Moreover, beyond their diagnostic value, bedside skills foster trust, communication, and rapport between patients and clinicians—essential elements of shared decision-making and patient-centered care.

To address this, medical training must emphasize a balanced approach, ensuring that clinicians integrate technological insights with sound clinical judgment for optimal patient management. To accomplish this, PE training —regardless of the teaching format—and other bedside skills must be reevaluated and given greater importance.^11^PE remains a fundamental clinical tool that, beyond its invaluable contribution to understanding physiopathology, can aid in decision-making, enhance efficiency, and strengthen the patient-physician relationship.

Ultimately, rather than viewing technological advancements and bedside skills as opposing forces, the medical community must strive for an integrated perspective, free from dogmatic conceptions about both the merits and shortcomings of each. Reinventing PE in medical education and daily practice will ensure that clinicians harness the best of both worlds—leveraging modern technology while preserving the irreplaceable value of hands-on assessment.
